# The detrimental effects of student-disordered behavior at school: evidence from using the cusp catastrophe

**DOI:** 10.3389/fpsyg.2023.1346232

**Published:** 2024-01-11

**Authors:** Ghadah Alkhadim

**Affiliations:** Faculty of Educational Psychology, Taif University, Taif, Saudi Arabia

**Keywords:** reading engagement, liking of reading, disordered behavior, cusp catastrophe, nonlinear modeling, nonlinear dynamics systems theory

## Abstract

**Introduction:**

The purpose of the present study is to examine the potentially complex relationship between disordered behavior at school and students’ engagement with reading activities given that they enjoy reading. Of particular interest is the role of disordered behavior which we believe moderated the relationship between liking reading and reading engagement.

**Methods:**

Participants were 2,420 fourth graders who participated in the 2021 PIRLS study from Saudi Arabia and were selected using stratified random sampling from 117 schools in the Kingdom. Data were analyzed using linear and nonlinear means such as the linear model, the logistic model, and the cusp catastrophe.

**Results:**

Results pointed to the superiority of the cusp catastrophe towards predicting student engagement in reading by highlighting the splitting role of students’ disruptive classroom behavior.

**Discussion:**

It was evident that exceeding a critical upward level in disruptive classroom behavior was associated with unpredictable and sudden changes in reading engagement. It is concluded that the application of non-linear means may be conducive to understanding complex educational phenomena.

## 1 Introduction

Students’ attitudes about reading are significantly impacted by the concept of engagement in reading, which includes behavioral, emotional, and cognitive components (Fredricks et al., 2004). According to Guthrie and Klauda (2008), this concept is a crucial factor in shaping students’ attitudes toward reading. When it comes to reading, behavioral engagement refers to the active participation in reading activities, whereas emotional engagement comprises the subjective emotional reactions that are experienced when reading such as joy and intrinsic interest (Baker et al., 2000; Unrau and Schlackman, 2006). On the other side, cognitive engagement refers to the mental effort and investment that is made to master the content that is being read (Fredricks et al., 2004) and involves the use of deep processing and strategic reading behaviors such as previewing, visualizing, monitoring, making connections, synthesizing, and summarizing (Pressley and Afflerbach, 1995; Wigfield and Guthrie, 1997; Duke and Pearson, 2002; Afflerbach et al., 2008).

The scientific literature emphasizes the importance of reading enjoyment in influencing students’ engagement with reading (Martínez et al., 2008; Smith et al., 2012; Afflerbach et al., 2013; Mol and Jolles, 2014; Lim et al., 2015; Ho and Lau, 2018; Merga and Roni, 2018; Preece and Levy, 2018; Amiruddin, 2022; Bergen et al., 2022). Reading enjoyment has been consistently found to have a significant positive association with students’ reading performance and achievement in diverse populations and age groups (Clark, 2011; Shanahan and Lonigan, 2015; Ho and Lau, 2018). Concerning the association between engagement and enjoyment, in a meta-analysis of 52 studies, Guthrie and Wigfield (2000) reported a correlation of r = 0.74 which is large by any standards (e.g., Cohen, 1992). Additionally, fostering students’ enjoyment of reading is imperative to support continued reading engagement (Merga and Roni, 2018). It has been highlighted that poor attitudes towards reading can lead to disengagement from reading activities (Martínez et al., 2008; Amiruddin, 2022). Furthermore, students’ self-perception of reading ability and enjoyment of reading have been identified as strong correlates of reading achievement (Smith et al., 2012). The correlation between reading enjoyment and reading skills represents a reciprocal association, indicating that literacy skills fuel literacy enjoyment, and vice versa (Bergen et al., 2022). It is believed that this association is through increased engagement with reading activities (Hidi et al., 2006). For example, Allgood et al. (2012) conducted an experimental study to increase student engagement with reading and the outcome was significant gains in reading achievement. Shared reading in school has been associated with increasing student learning, engagement, motivation, and enjoyment (Merga, 2017). Moreover, when students are absorbed in the world of the book, they tend to be particularly engaged in their reading activity (Mol and Jolles, 2014). For example, Guthrie and Wigfield (2000) discovered that students who display elevated levels of engagement, specifically in terms of having the ability to choose and exercise autonomy in their reading activities, exhibit a stronger sense of enjoyment towards reading. This pleasurable experience, in turn, facilitates a positive feedback loop, strengthening the level of involvement (Mol and Bus, 2011).

### 1.1 Reading engagement and disruptive classroom behavior

Student’s disruptive behavior can make the classroom atmosphere unsuitable for learning (Stage and Quiroz, 1997). It may result in less time spent on teaching and divert the attention of other pupils, which might have an indirect negative impact on the class’s reading engagement and success (Sullivan et al., 2014). Research has demonstrated that the use of effective classroom management strategies is associated with a reduction in disruptive conduct and a corresponding improvement in academic performance (Bradshaw et al., 2010). Examples of good practices are peer-assisted learning Sinclair et al. (2019), the good behavior game (Smith et al., 2012), and the systematic analysis of behavior (Shumate and Wills, 2010). Furthermore, previous research has demonstrated that disruptive behaviors play a crucial role in moderating the relationship between class assignment and reading proficiency in kindergarten, pointing to the potentially detrimental effect on reading acquisition (Coventry et al., 2009). Even more important is the fact that disruptive behaviors have a substantial role in moderating the relationship between students’ reading proficiency, their conduct within the educational setting, and the instructional competencies of teachers (Brokamp et al., 2018). Research has demonstrated that the presence of disruptive behavior can lead to adverse psychological effects such as stress, anxiety, annoyance, and even rage on the part of teachers. Poor teacher-student relationships can result in lower expectations and lower-quality instruction, which can affect students’ reading success (Hughes et al., 2008). These negative emotions can hinder effective communication and collaboration among individuals, ultimately leading to a possible decline in the quality of education and services provided (Rosenstein and O’Daniel, 2008) and similarly significant decrements in students’ reading achievement (Pisecco et al., 2001). Interestingly, the potentially moderating role of a disruptive classroom environment on student engagement with reading activities has only been investigated using linear analytical means assuming an analogous effect across all levels of disruptive behavior. The present study hypothesizes that the relationship between disruptive student behaviors and engagement in reading activities is most likely non-linear and best described by the cusp catastrophe (Cobb and Zacks, 1985). Below there is an analytical account of this thesis.

### 1.2 Nonlinear dynamics and the cusp catastrophe model

The cusp catastrophe model, a fundamental idea within the field of nonlinear dynamical systems theory, was formulated by René Thom in the 1970s and subsequently popularized by Eric Zeeman. This model plays a crucial role in explaining abrupt and profound shifts in behavior or occurrences, which linear models encounter difficulty in accurately forecasting. The extensive utilization of the cusp catastrophe model across many fields highlights its adaptability and a broad range of applications. The economic model elucidates non-linear associations between predictors and outcomes, encompassing the dynamics of financial markets during times of crisis and the anticipation of pivotal junctures within economic systems (Chen et al., 2014, 2020). Within the field of engineering, the utilization of stability analysis is prevalent in the examination of nonlinear material structures and the anticipation of catastrophic failures resulting from stress-induced conditions (Wang et al., 2011). Furthermore, the utilization of the model has been observed in the domains of public health, and behavioral research, as well as in the comprehension of intricate phenomena such as the dynamics of rangeland ecosystems and fetal heart rate decelerations (Lockwood and Lockwood, 1993; Kikuchi et al., 2006). More recently, several studies in education, educational psychology, and mainstream psychology have employed the cusp catastrophe model. These studies attempted to explain the roles and functioning of motivation (Stamovlasis and Gonida, 2018), problem solving (Stamovlasis and Tsaparlis, 2012), health (Clair, 1998) or public health concerns (Ding-Geng and Chen, 2017) to mention a few.

The functioning of the cusp model is based on the integration of two control parameters, which serve as external factors affecting the system, along with a behavior variable that signifies the current state of the system. As the aforementioned parameters exhibit variability, the system experiences a significant metamorphosis, distinguished by an abrupt transition from one state to another. The sudden transition, referred to as a cusp., takes place along a distinct curve inside the parameter space, highlighting the significant influence of these external inputs in initiating the system’s metamorphosis. The model is represented as a three-dimensional surface, frequently exhibiting a cusp-like form, wherein smooth variations in the control parameters can result in sudden and discontinuous alterations in the behavior variable, a phenomenon referred to as ‘bifurcation’. In equation form (Lockwood and Lockwood, 1993), the cusp catastrophe model is described by a potential function *V* (*y*, *a*, *b*) as follows:


(1) $$V\left(y\alpha \beta \right)=\alpha y+\frac{1}{2}\beta y^{2}-\frac{1}{4}y^{4}$$

With the potential function ‘*V*’, state variable ‘*y*’, and the asymmetry and bifurcation parameters ‘*a*’ and ‘*b*’. The values of the parameters ‘*a*’ and ‘*b*’, which are considered to move slowly in comparison to *y*, define the state of the system. As the two control parameters change the behavior evolves either gradually or suddenly depending on when the bifurcation term value enters the so-called critical point for which abrupt and sudden changes in the outcome variable in any direction are expected.

The purpose of the present study was to explore the potentially complex relationship between disordered behavior at school and students’ engagement with reading activities given that they enjoy reading. Of particular interest is the role of disordered behavior which we believe moderated the relationship between liking reading and reading engagement. It is hypothesized that its role is moderating but also in a non-linear fashion. That is, moderators are evaluated at different levels within their linear scaling. For disordered and disruptive behavior in the class, this relationship is likely non-linear as reading engagement likely drops to extremely low levels when disruption levels exceed any manageable by the teacher level. Consequently, the relationship between student-disordered behavior in the class and engagement with reading activities is likely better modeled within the cusp catastrophe model for which engagement may likely present itself with abrupt and discontinuous alterations.

## 2 Method

### 2.1 Participants

Participants were 2,420 fourth graders who participated in the 2021 PIRLS study from Saudi Arabia. Students were selected using stratified random sampling from 117 schools in the Kingdom. Only Saudi students and those who had complete data participated, thus, listwise deletion was employed. Exclusionary criteria involved international students or students whose native language was not Arabic and those whose achievement was too low to be estimated to avoid floor effects in achievement. There were 1,434 girls (59.3%) and 986 boys (40.7%). Data, methodology, scales, and reports from PIRLS 2021 may be accessed directly at: https://pirls2021.org/.

### 2.2 Measures

All scales were completed by students. Estimation of internal consistency reliability involved Cronbach’s alpha.

#### 2.2.1 Disorderly behavior during lessons

This scale is comprised of five items evaluating the frequency with which disorderly conduct is present in the classroom and is based on student reports. Example behaviors were “students do not listen to what the teacher says” or “there is too much noise for students to work well.” (see Supplementary Appendix A). Items were scored using a 4-point rating scale system anchored between the options “never” and “every or almost every lesson.” The scale was utilized using its original scoring system which was based on the fit of the Rasch model. The direction of scoring was so that lower scores are indicative of aberrant behavioral patterns. Alpha internal consistency reliability was 0.83.

#### 2.2.2 Students like reading

This scale also completed by students was comprised of 8 items utilizing a r-point scaling system denoting agreement to disagreement. Item content related to the joy of reading, the challenge and learning from reading, etc. (see Supplementary Appendix A). The scale scores using the Rasch model were utilized as per the developer’s suggestions. Higher scores were indicative of higher interest and joy from being engaged in reading activities. Alpha internal consistency reliability was 0.81.

#### 2.2.3 Students engaged in reading lessons

This scale included nine items using a 4-point agreement-disagreement scaling system. Sample items were “My teacher gives me interesting things to read,” and “My teacher encourages me to say what I think about what I have read” (see Supplementary Appendix A). Higher scores were indicative of higher engagement with reading tasks. The alpha internal consistency reliability of the scale was 0.83.

### 2.3 Data analyzes

#### 2.3.1 Cusp catastrophe model and prerequisite assumptions

The main assumption of the cusp model is the presence of bimodality or multimodality in the dependent variable suggesting different states of behavior as a function of the asymmetry and bifurcation variables. For this reason, I employed the multimode package (Ameijeiras-Alonso et al., 2021) in R which acts as a toolbox for assessing multimodality by engaging the diptest package (Maechler, 2016) for applying the Hartigan and Hartigan (1985) procedure, and the modeest package (Poncet, 2019) to assess the true number of modes.

**Figure 1:**
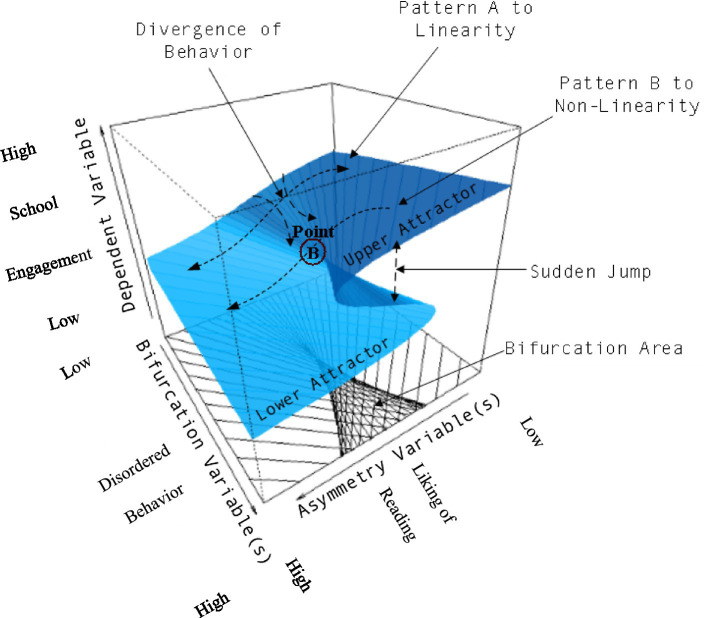
Description of the cusp model within the context of student’s engagement with reading activities (outcome variable) predicted by the linear effects of students’ liking of reading (asymmetry variable) and the splitting effects from having a disordered classroom environment (bifurcation variable).

The cusp catastrophe model was evaluated using the cusp package in R (Grasman et al., 2009) and variables were standardized as theta scores from the Rasch model were used. Figure 1 displays the main theses of the cusp catastrophe in the context of students’ engagement in reading. When levels in the asymmetry variable (namely liking of reading) and the bifurcation variable (disordered behavior in the classroom) are low, the relationship between student reading engagement and disruptive behavior is likely linear and positive as shown in Pattern A. However, when levels of disruption exceed a critical high level, termed the cusp point (point B in the figure), from which the classroom environment is no longer conducive to learning, the cusp model expects that reading engagement becomes unpredictable and is no longer explained using linear terms (see Pattern B). This qualitative description of the reading engagement process provided by its three-dimensional model renders it a potent tool for understanding complex and multivariate educational phenomena (Chen and Chen, 2015). Omnibus model fit was evaluated by contrasting the cusp model with the linear model (as in multiple regression analysis with all predictors entered in one step), and the logistic model (evaluating the behavior of the outcome variable using an S-shaped curve). In particular, the logistic model provides for a competing alternative to the cusp model as it also models nonlinear trajectories. The level of significance was set to 0.01 to account for the relatively large sample size and the correspondingly large amounts of statistical power.

## 3 Results

### 3.1 Prerequisite statistical analyzes

**Figure 2:**
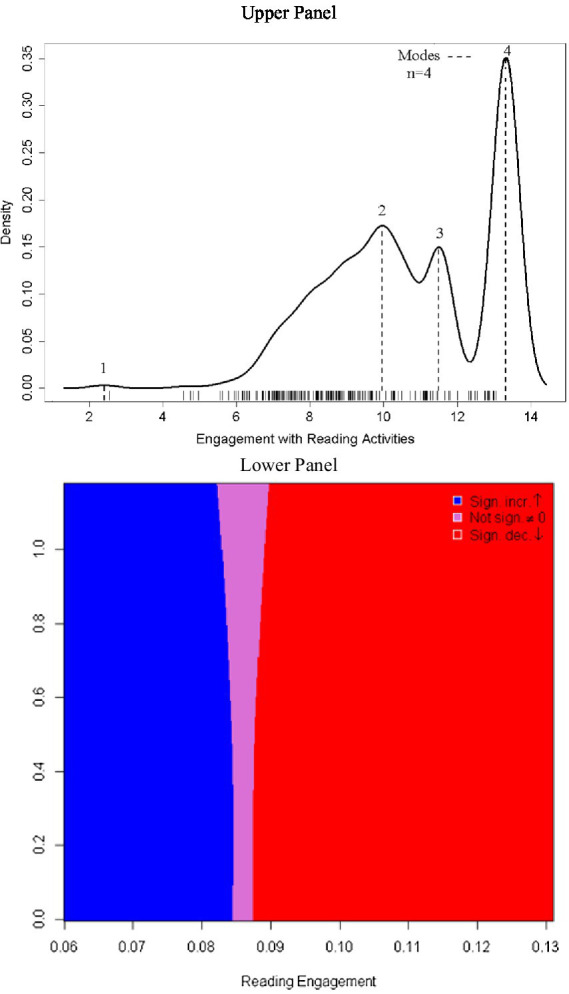
Multimodality in student engagement with reading activities at school using density plot (upper panel) and the sizer map (lower panel).

Figure 2 displays the findings from the tests of bimodality and multimodality. As shown in Figure 2, upper panel, four modes were identified. First, the conclusion of multimodality was confirmed using Hartigan’s dip test for unimodality (D = 0.084, *p* < 0.001). As a second step, Silverman’s (1981) critical bandwidth test evaluated alternative hypotheses for the presence of more than one mode. All tests up to 3 modes pointed to accepting the alternative hypothesis that a different number of modes was evident. Only when 4 modes was the reference value, the null hypothesis was supported in that the actual number of modalities was not different from four (Critical bandwidth = 0.363, *p* = 0.058). Figure 2, lower panel, displays the sizer plot with the transition between the colors blue and purple indicating a change in the trajectory of behavior from a negative trend to a zero trend, and colors transitioning from purple to red, changes in behavior from a zero trend to a positive trend (Chaudhuri and Marron, 1999). All this information adds evidence to the conclusion of multimodality in the dependent variable.

### 3.2 Prediction of reading engagement from reading enjoyment and a disordered classroom environment

Table 1 displays global fit statistics from contrasting linear, logistic, and cusp models. As shown in the table, all information criteria values were saliently smaller in the cusp model compared to the linear and logistic comparison models. Further evidence was provided by contrasting the linear and cusp model using a chi-square difference test, which was significant in favor of the latter [*χ*^2^ (3) = 4,698, *p* < 0.001]. Thus, model fit significantly favored the cusp model over competing models.

**Table 1:** Model comparison across linear and cusp models using descriptive information criteria.

Models tested	Loglikelihood	Parameters	AIC	AICc	BIC
1. Linear	−5836.003	4	11680.010	11680.020	11703.725
2. Logistic	−5776.694	5	11563.390	11563.410	11593.038
3. Cusp	−3486.770	7	6987.540	6987.581	7029.049

Npar, Number of estimated parameters; AIC, Akaike criterion; AICc, Corrected AIC with an adjustment for sample size; BIC, Bayesian information criterion.

Table 2 displays the parameters of the cusp model, with all being significantly different from zero. Focusing on the slope terms of the asymmetry and bifurcation variables, the liking of reading was a significant positive predictor of student engagement with reading as expected (*b* = 0.331, *p* < 0.001). Similarly, student disordering in the classroom had a positive slope which is associated with the presence of sudden and unpredictable changes in reading engagement as per the cusp model premises (*b* = 0.112, *p* < 0.001). Thus, as the asymmetry factor increases, that is, the liking of reading and disordered behavior is at low levels student engagement with reading grows linearly. However, when classroom-disordered behavior grows beyond some critical adaptive point, student engagement with reading takes on various values and becomes unpredictable.

**Table 2:** Parameter estimates of the cusp model for the prediction of student engagement with reading activities as a function of student liking of reading (asymmetry var.) and disordered student behavior in class (bifurcation var.).

Terms in Cusp Model	Slope	LCI_95%_	UCI_95%_	S.E.	*Z*-test	*p*-value
a_0_(Intercept)	−3.015	−3.681	−2.348	0.340	−8.859	<0.001***
a_1_(Liking of Reading)	0.331	0.276	0.387	0.028	11.74	<0.001***
b_0_(Intercept)	−1.453	−1.550	−1.356	0.050	−29.272	<0.001***
b_1_(Student Disordered Behavior)	0.112	0.103	0.121	0.005	24.127	<0.001***
w_0_(Intercept)	−3.522	−3.639	−3.405	0.060	−59.004	<0.001***
w_1_(Student Engagement)	0.364	0.356	0.373	0.004	82.067	<0.001***

The terms a, b, and w refer to asymmetry, bifurcation, and outcome variables’ intercept and slope terms, respectively; Intercepts are denoted with the ‘0’ subscript and slopes with “1”.

LCI_95%_ = Lower 95% Confidence Interval; UCI_95%_ = Lower 95% Confidence Interval.

***p < 0.001, **p < 0.01, *p < 0.05 for two-tailed tests. The observed *p*-values were further corrected for experimenter-wise error and were still all significant at *p* < 0.001.

**Figure 3:**
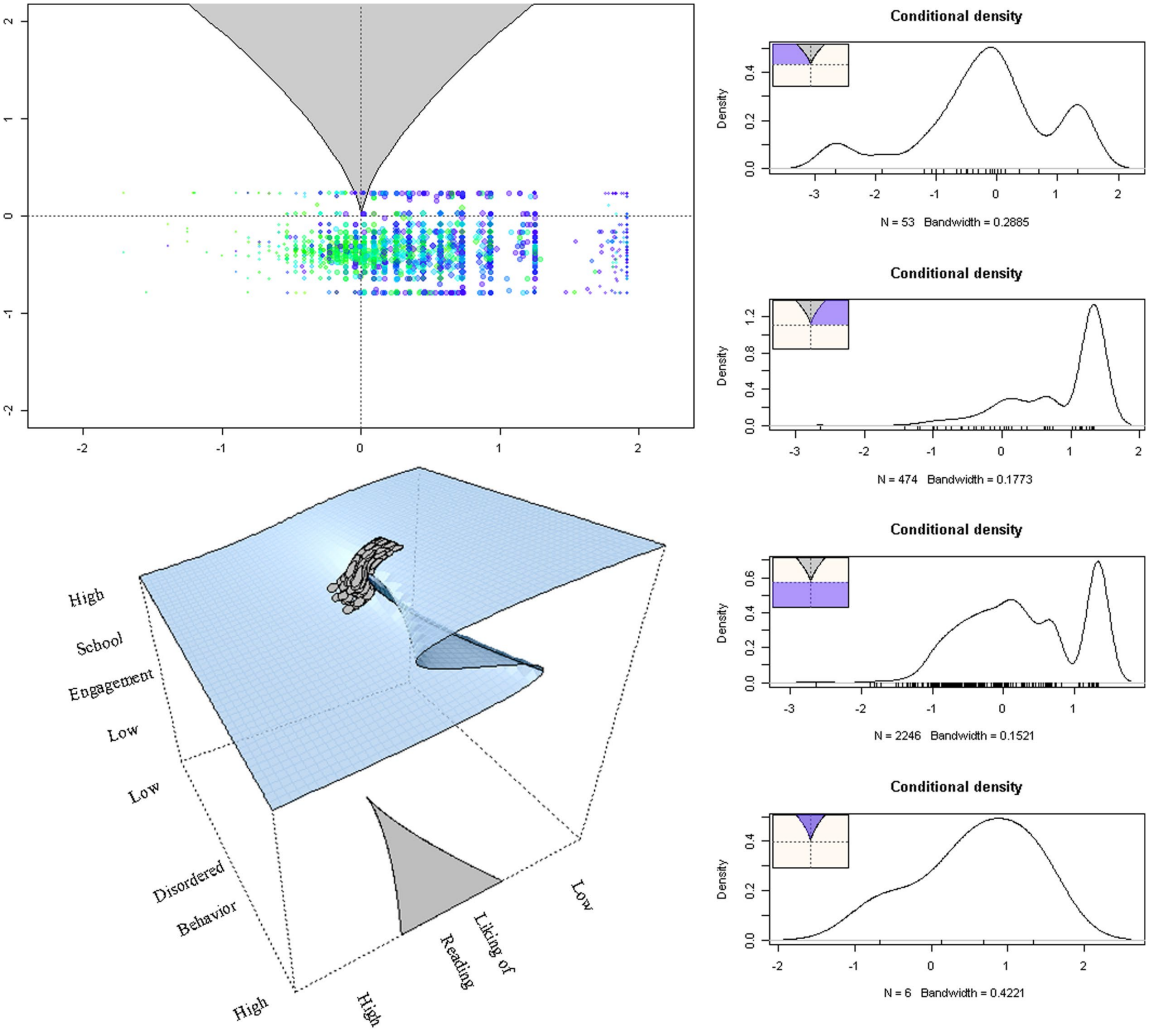
The upper left figure displays the lower surface with observations oscillating from the upper to the lower surface. The lower left figure shows observations transitioning across surfaces. The figures to the right show densities at various locations on the response surface. The terms “High” and “Low” refer to levels of the outcome, asymmetry, and bifurcation variables.

**Figure 4:**
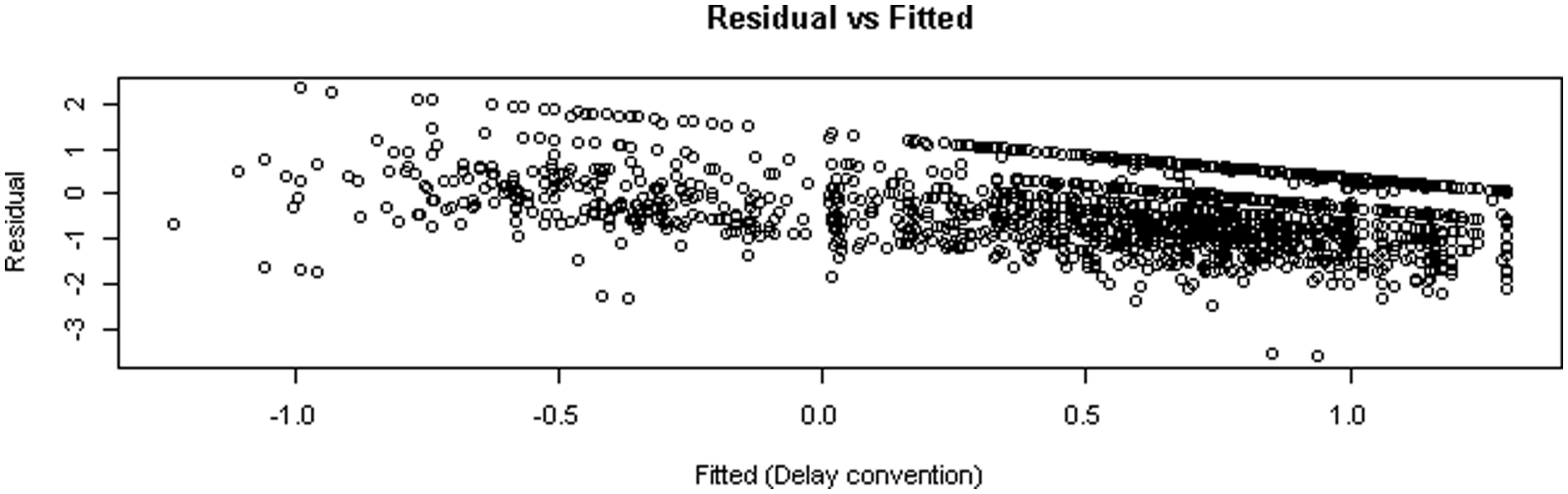
Scatterplot of fitted vs. residual values in the cusp catastrophe model.

Figure 3, right panel, displays distributions of students’ responses at various areas of the lower response surface. As posited by the main theses of the model (e.g., Cobb and Zacks, 1985), bimodality and multimodality are evident at various areas within the response surface with a small number of observations (i.e., *n* = 6) being present within the bifurcation area. The upper left panel of Figure 3 displays the observations as they oscillate from the upper to the lower surface. Observations with “darker” colors are closer to the upper surface and the opposite is true of observations with lighter colors. The larger dots are indicative of coordinates with data from more than one participant. The lower right part of the figure displays the observations as they move from the upper to the lower surface. Last, Figure 4 displays residual versus fitted values for which a slight negative trend is to be expected as was the case with simulated data (Grasman et al., 2009). Collectively all the information corroborates with the idea that the present data were a good fit for the cusp catastrophe model.

## 4 Discussion

The purpose of the present study was to explore the potentially complex relationship between disordered behavior at school and students’ engagement with reading activities given that they like and enjoy reading. Of particular interest is the role of disordered behavior which, as expected, moderated the relationship between students’ liking of reading and reading engagement.

With reading enjoyment serving as the asymmetry variable and disordered student behavior as the bifurcation variable, the cusp catastrophe model offered a sophisticated knowledge of how engagement can fluctuate suddenly and unexpectedly due to the classroom environment. The prevailing scenario from the present findings is that engagement remains stable when disruptive behavior in the classroom increases up to some moderate levels that define a critical point, the cusp point. Beyond that point, any minor increase in students’ disruptive behavior is likely associated with a significant and sudden drop in students’ engagement with reading activities. This finding adds to the scientific literature that has demonstrated the negative propensities of disruptive behavior in the classroom and suggests tha these effects are more pronounced than what was earlier predicted using the linear model (Stage and Quiroz, 1997; Bradshaw et al., 2010). Empirical studies conductedin educational settings have yielded evidence that supports the presence of nonlinear effects in student engagement (Oliver et al., 2011). For example, scientific studies have demonstrated that levels of engagement can vary significantly and are influenced by factors such as students’ positive emotions and adaptive coping strategies (Reschly et al., 2008; Guardino and Fullerton, 2010; Cook et al., 2013). The present results highlight the significance of maintaining a well-structured classroom environment and cultivating a favorable mindset towards reading. Even minor adjustments in these aspects can result in notable improvements in student involvement. This method emphasizes how important it is for the classroom environment and each student’s attitudes toward reading to play a part in determining engagement patterns. This approach provides educators with a framework for recognizing and resolving the critical elements that might have an abrupt effect on students’ participation in academic activities.

### 4.1 Study limitations and future directions

Several items related to the cusp catastrophe model contribute to its limitations. First, causality cannot be inferred as a correlational design was utilized and the data represent a snapshot of what was in place during 2021 in schools in the Saudi Arabia Kingdom. Second, the cusp model has been criticized for lacking generalizability as individual and contextual factors vary by classroom and school, thus, the generality of the present findings should be viewed with caution (see Cobb and Zacks, 1985). Third, overfitting the model is a potential risk as simpler analytical models may also fit the specific model and be preferred using the principle of parsimony (Stewart, 1981). Fourth, the self-reported nature of the data are associated with some degree of correlation due to the common method. Thus, the observed relationships may be likely inflated due to medium. Last, the analytical methodology reflected a selection of Cobb’s model among other alternatives such as Guastello’s polynomial regression. In the future, it will be important to replicate the present findings and extend them by including person-relevant attributes that may act as a buffer against the negative effects of a disruptive classroom environment. Gender differences and social-contextual factors such as student SES and private or public schooling may be important moderators towards understanding such complex educational phenomena.

## Data availability statement

Publicly available datasets were analyzed in this study. This data can be found at: https://pirls2021.org/.

## Ethics statement

Ethical approval was not required for the study involving humans in accordance with the local legislation and institutional requirements. Written informed consent to participate in this study was not required from the participants or the participants' legal guardians/next of kin in accordance with the national legislation and the institutional requirements.

## Author contributions

GA: Conceptualization, Data curation, Formal analysis, Methodology, Validation, Visualization, Writing – original draft, Writing – review & editing.

## References

[ref1] Afflerbach P. Cho B. Kim J. Crassas M. Doyle B. (2013). Reading: what else matters besides strategies and skills? Read. Teach. 66, 440–448. doi: 10.1002/TRTR.1146

[ref2] Afflerbach P. Pearson P. D. Paris S. G. (2008). Clarifying differences between reading skills and reading strategies. Read. Teach. 61, 364–373. doi: 10.1598/RT.61.5.1

[ref3] Allgood J. Welch M. B. Wenner J. (2012). An examination of the effects of a reading intervention that incorporates student engagement. Elem. Sch. J. 113, 57–82.

[ref4] Ameijeiras-Alonso J. Crujeiras R. M. Rodríguez-Casal A. (2021). Multimode: Mode testing and exploring. R package version 1.5. Available at: https://CRAN.R-project.org/package=multimode

[ref5] Amiruddin A. (2022). The influence of sq3r technique and students’ reading interest towards students’ reading comprehension achievement. J. Soc. Work Educ. 3, 60–66. doi: 10.52690/jswse.v3i1.273

[ref6] Baker L. Dreher M. J. Guthrie J. T. (Eds.). (2000). Engaging young readers: Promoting achievement and motivation. Guilford Press. New York, NY

[ref7] Bergen E. Hart S. Latvala A. Vuoksimaa E. Tolvanen A. Torppa M. (2022). Literacy skills seem to fuel literacy enjoyment, rather than vice versa. Dev. Sci. 26:e13325. doi: 10.1111/desc.13325, PMID: 36101942 PMC10008752

[ref8] Bradshaw C. P. Mitchell M. M. Leaf P. J. (2010). Examining the effects of schoolwide positive behavioral interventions and supports on student outcomes. J. Posit. Behav. Interv. 12, 133–148. doi: 10.1177/1098300709334798

[ref9] Brokamp S. Houtveen A. Grift W. (2018). The relationship among students' reading performance, their classroom behavior, and teacher skills. J. Educ. Res. 112, 1–11. doi: 10.1080/00220671.2017.1411878

[ref10] Chaudhuri P. Marron J. S. (1999). SiZer for exploration of structures in curves. J. Am. Stat. Assoc. 94, 807–823. doi: 10.1080/01621459.1999.10474186

[ref9004] Chen D. G. Lin F. Chen X. Tang W. (2014). Cusp catastrophe model: A nonlinear model for health outcomes in nursing research. Nurs. Res. 63, 211–220. 24785249 10.1097/NNR.0000000000000034 PMC4066972

[ref9003] Chen X. Chen D. (2015). “Cusp catastrophe modeling in medical and health research,” in Innovative statistical methods for public health data. eds. Chen D.-G. Wilson J. (Cham, Switzerland: Springer).

[ref9005] Chen X. Wang K. Chen D. G. (2020). “Cusp catastrophe regression analysis of testosterone in bifurcating the age-related changes in PSA, a biomarker for prostate cancer,” in Statistical methods for global health and epidemiology: Principles, methods and applications. eds. Chen X. Chen D. G. (Cham, Switzerland: Springer), 353–372.

[ref11] Clair S. (1998). A cusp catastrophe model for adolescent alcohol use: an empirical test. Nonlinear Dyn. Psychol. 2, 217–241. doi: 10.1023/A:1022376002167

[ref12] Clark C. M. (2011). Reading enjoyment and motivation: a complex relationship of literacy and socio-emotional factors. Read. Res. Q. 46, 315–339.

[ref13] Cobb L. Zacks S. (1985). Applications of catastrophe theory for statistical modeling in the biosciences. J. Am. Stat. Assoc. 80, 793–802. doi: 10.1080/01621459.1985.10478184

[ref14] Cohen J. (1992). A power primer. Psychol. Bull. 112, 155–159. doi: 10.1037/0033-2909.112.1.155, PMID: 19565683

[ref15] Cook C. Collins T. Dart E. Vance M. McIntosh K. Grady E.. (2013). Evaluation of the class pass intervention for typically developing students with hypothesized escape-motivated disruptive classroom behavior. Psychol. Sch. 51, 107–125. doi: 10.1002/pits.21742

[ref16] Coventry W. Byrne B. Coleman M. Olson R. Corley R. Willcutt E.. (2009). Does classroom separation affect twins' reading ability in the early years of school? Twin Res. Hum. Genet. 12, 455–461. doi: 10.1375/twin.12.5.455, PMID: 19803773 PMC3915871

[ref17] Ding-Geng C. Chen X. (2017). Cusp catastrophe regression and its application in public health and behavioral research. Int. J. Environ. Res. Public Health 14:1220. doi: 10.3390/ijerph14101220, PMID: 29027967 PMC5664721

[ref18] Duke N. K. Pearson P. D. (2002). “Effective practices for developing reading comprehension” in What research has to say about reading instruction. eds. Farstrup A. E. Samuels S. J.. 3rd ed (Newark, DE: International Reading Association), 205–242.

[ref19] Fredricks J. A. Blumenfeld P. C. Paris A. H. (2004). School engagement: potential of the concept, state of the evidence. Rev. Educ. Res. 74, 59–109. doi: 10.3102/00346543074001059

[ref9007] Grasman R. P. van der Maas H. L. Wagenmakers E. J. (2009). Fitting the cusp catastrophe in R: A cusp-package primer. J. Stat. Softw. 32, 1–27.

[ref20] Guardino C. Fullerton E. (2010). Changing behaviors by changing the classroom environment. Teach. Except. Child. 42, 8–13. doi: 10.1177/004005991004200601

[ref21] Guthrie J. T. Klauda S. L. (2008). “Engagement and motivation in reading” in Handbook of reading research. eds. Farstrup A. Samuels S. J., vol. 4 (Abingdon: Taylor & Francis), 408–432.

[ref22] Guthrie J. T. Wigfield A. (2000). “Engagement and motivation in reading” in Handbook of reading research. eds. Kamil M. L. Mosenthal P. B. Pearson P. D. Barr R., vol. III (Mahwah, NJ: Lawrence Erlbaum Associates), 403–422.

[ref23] Hartigan J. A. Hartigan P. M. (1985). The dip test of unimodality. Ann. Stat. 13, 70–84. doi: 10.1214/aos/1176346577

[ref24] Hidi S. Berndt K. Pearson A. D. (2006). Is interest important? A meta-analysis of interest in learning and its effects on student motivation and achievement. Rev. Educ. Res. 76, 123–156.

[ref25] Ho E. Lau K. (2018). Reading engagement and reading literacy performance: effective policy and practices at home and in school. J. Res. Read. 41, 657–679. doi: 10.1111/1467-9817.12246

[ref26] Hughes J. N. Luo W. Kwok O. M. Loyd L. K. (2008). Teacher-student support, effortful engagement, and achievement: a 3-year longitudinal study. J. Educ. Psychol. 100, 1–14. doi: 10.1037/0022-0663.100.1.1, PMID: 19578558 PMC2705122

[ref9001] Kikuchi A. Unno N. Horikoshi T. Kozuma S. Taketani Y. (2006). Catastrophe theory model for decelerations of fetal heart rate. Gynecol. Obstet. Invest. 61, 72–9. doi: 10.1159/000088812 16210855

[ref27] Lim H. Bong M. Woo Y. (2015). Reading attitude as a mediator between contextual factors and reading behavior. Teach. Coll. Rec. 117, 1–36. doi: 10.1177/016146811511700116

[ref9002] Lockwood J. Lockwood D. R. (1993). Catastrophe theory: A unified paradigm for rangeland ecosystem dynamics. J. Range Manag. 46, 282–288.

[ref28] Maechler M. (2016). Diptest: Hartigan’s dip test statistic for unimodality – Corrected. R package version 0.75–7. Available at: http://CRAN.R-project.org/package=diptest

[ref29] Martínez R. Arıcak O. Jewell J. (2008). Influence of reading attitude on reading achievement: a test of the temporal-interaction model. Psychol. Sch. 45, 1010–1023. doi: 10.1002/pits.20348

[ref30] Merga M. (2017). Interactive reading opportunities beyond the early years: what educators need to consider. Aust. J. Educ. 61, 328–343. doi: 10.1177/0004944117727749

[ref31] Merga M. Roni S. (2018). Empowering parents to encourage children to read beyond the early years. Read. Teach. 72, 213–221. doi: 10.1002/trtr.1703

[ref32] Mol S. E. Bus A. G. (2011). To read or not to read: a meta-analysis of print exposure from infancy to early adulthood. Psychol. Bull. 137, 267–296. doi: 10.1037/a0021890 21219054

[ref33] Mol S. Jolles J. (2014). Reading enjoyment amongst non-leisure readers can affect achievement in secondary school. Front. Psychol. 5:1214. doi: 10.3389/fpsyg.2014.01214, PMID: 25386154 PMC4209810

[ref34] Oliver R. Wehby J. Reschly D. (2011). Teacher classroom management practices: effects on disruptive or aggressive student behavior. Campbell Syst. Rev. 7, 1–55. doi: 10.4073/csr.2011.4

[ref35] Pisecco S. Wristers K. Swank P. Silva P. Baker D. (2001). The effect of academic self-concept on adhd and antisocial behaviors in early adolescence. J. Learn. Disabil. 34, 450–461. doi: 10.1177/002221940103400506, PMID: 15503593

[ref36] Poncet P. (2019). Modeest: mode estimation. R package version 2.4.0. Available at: https://CRAN R-project.org/package=modeest

[ref37] Preece J. Levy R. (2018). Understanding the barriers and motivations to shared reading with young children: the role of enjoyment and feedback. J. Early Child. Lit. 20, 631–654. doi: 10.1177/1468798418779216

[ref38] Pressley M. Afflerbach P. (1995). Verbal protocols of reading: The nature of constructively responsive reading. Hillsdale, NJ: Erlbaum.

[ref39] Reschly A. L. Huebner E. S. Appleton J. J. Antaramian S. (2008). Engagement as flourishing: the contribution of positive emotions and coping to adolescents' engagement at school and with learning. Psychol. Sch. 45, 419–431. doi: 10.1002/pits.20306

[ref40] Rosenstein A. O’Daniel M. (2008). Invited article: managing disruptive physician behavior: impact on staff relationships and patient care. Neurology 70, 1564–1570. doi: 10.1212/01.wnl.0000310641.26223.82, PMID: 18427073

[ref41] Shanahan T. Lonigan C. J. (2015). Effective reading instruction for young children: Kindergarten through 3rd grade (2nd). Guilford Press. New York, NY

[ref42] Shumate E. Wills H. (2010). Classroom-based functional analysis and intervention for disruptive and off-task behaviors. Educ Treat Child 33, 23–48. doi: 10.1353/etc.0.0088

[ref9006] Silverman B. W. (1981). Using kernel density estimates to investigate multimodality. J. R. Stat. Soc. Series B 43, 97–99.

[ref43] Sinclair A. Gesel S. Lemons C. (2019). The effects of peer-assisted learning on disruptive behavior and academic engagement. J. Posit. Behav. Interv. 21, 238–248. doi: 10.1177/1098300719851227

[ref44] Smith J. Smith L. Gilmore A. Jameson M. (2012). Students' self-perception of reading ability, enjoyment of reading and reading achievement. Learn. Individ. Differ. 22, 202–206. doi: 10.1016/j.lindif.2011.04.010

[ref45] Stage S. A. Quiroz D. R. (1997). A meta-analysis of interventions to decrease disruptive classroom behavior in public education settings. Sch. Psychol. Rev. 26, 333–368. doi: 10.1080/02796015.1997.12085871

[ref46] Stamovlasis D. Gonida E. (2018). Dynamic effects of performance-avoidance goal orientation on student achievement in language and mathematics. Nonlinear Dynamics Psychol. Life Sci. 22, 335–358. PMID: 29908058

[ref47] Stamovlasis D. Tsaparlis G. (2012). Applying catastrophe theory to an information-processing model of problem solving in science education. Sci. Educ. 96, 392–410. doi: 10.1002/sce.21002

[ref48] Stewart I. (1981). Catastrophe theory in psychology: a user's guide. Int. J. Man-Mach. Stud. 14, 385–399.

[ref49] Sullivan A. L. Van Norman E. R. Klingbeil D. A. (2014). Exclusionary discipline of students with disabilities: student behaviors and school characteristics. J. Emot. Behav. Disord. 22, 212–222.

[ref50] Unrau N. Schlackman J. (2006). Motivation and its relationship with reading achievement in an urban middle school. J. Educ. Res. 100, 81–101. doi: 10.3200/JOER.100.2.81-101

[ref9008] Wang T. T. Yan X. Z. Yang H. L. Yang X. J. (2011). Stability analysis of the pillars between bedded salt cavern gas storages by cusp catastrophe model. Sci. China Technol. Sci. 54, 1616–1623.

[ref51] Wigfield A. Guthrie J. T. (1997). Relations of children's motivation for reading to the amount and breadth of their reading. J. Educ. Psychol. 89, 420–432. doi: 10.1037/0022-0663.89.3.420

