# Supplementary material for: The detrimental effects of student-disordered behavior at school: evidence from using the cusp catastrophe
Source: Front Psychol. 2024 Jan 11;14:1346232. doi: 10.3389/fpsyg.2023.1346232 (PMC10808790; doi:10.3389/fpsyg.2023.1346232)
Supplement: Supplementary file 1 [file Data_Sheet_1.docx]

**Appendix A**

*Scales from PIRLS 2021 that were utilized in the present study.*

| **Disorderly Behavior During Reading Lessons**  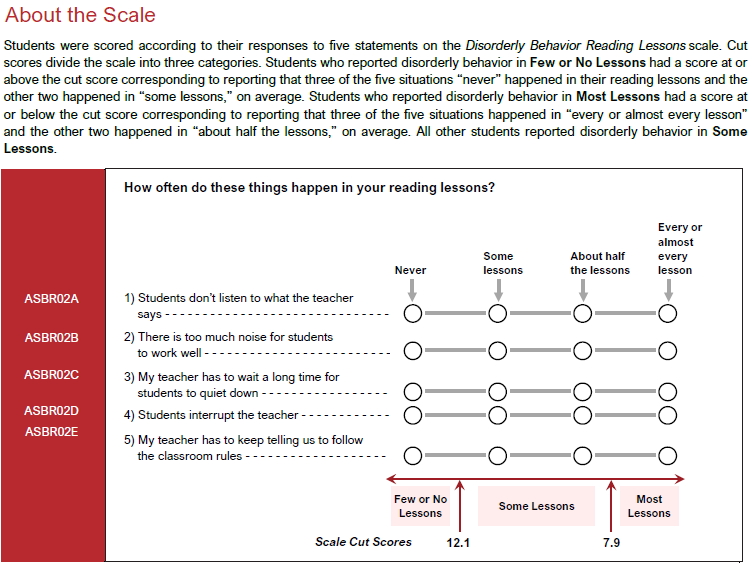 |
| --- |

| **Students Like Reading**  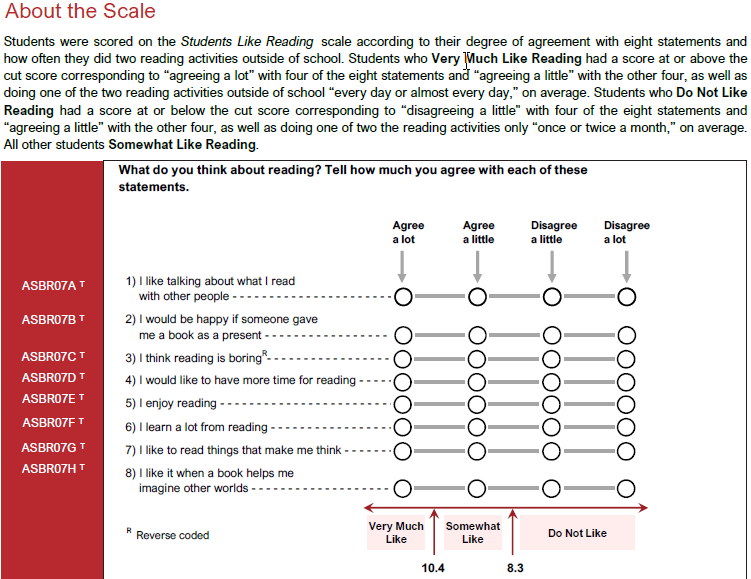 |
| --- |

| **Students Engaged in Reading Lessons**  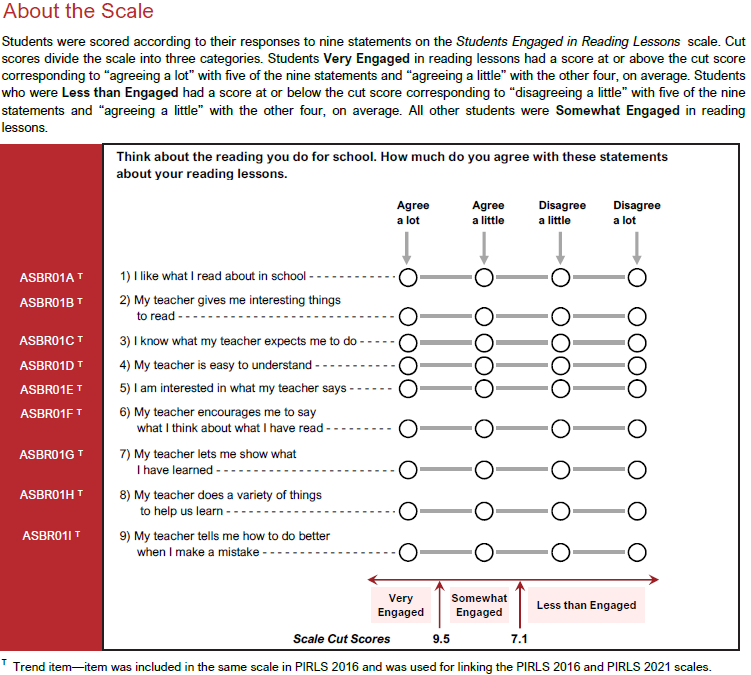 |
| --- |
